# Antioxidants and Exercise Performance: Focus on Mediterranean Diet

**DOI:** 10.3390/antiox15010010

**Published:** 2025-12-21

**Authors:** Giuseppe Annunziata, Elisabetta Camajani, Martina Galasso, Ludovica Verde, Massimiliano Caprio, Giovanna Muscogiuri, Antonio Paoli, Luigi Barrea

**Affiliations:** 1 Dipartimento di Psicologia e Scienze Della Salute, Università Telematica Pegaso, Centro Direzionale, Via Porzio, Isola F2, 80143 Naples, Italy; giuseppe.annunziata@unipegaso.it; 2 Department for the Promotion of Human Sciences and Quality of Life, San Raffaele Roma Open University, 00166 Rome, Italy; elisabetta.camajani@uniroma5.it (E.C.); massimiliano.caprio@sanraffaele.it (M.C.); 3 Laboratory of Cardiovascular Endocrinology, IRCCS San Raffaele, 00166 Rome, Italy; 4 Unità di Endocrinologia, Dipartimento di Medicina Clinica e Chirurgia, University of Naples Federico II, 80131 Naples, Italy; marti.galasso@studenti.unina.it; 5 Department of Public Health, University of Naples Federico II, Via Sergio Pansini 5, 80131 Naples, Italy; ludovica.verde@unina.it; 6 Centro Italiano per la Cura e il Benessere del Paziente con Obesità (C.I.B.O), Dipartimento di Endocrinologia, Diabetologia, Andrologia e Nutrizione, AOU Federico II, Via Sergio Pansini 5, 80131 Naples, Italy; giovanna.muscogiuri@unina.it; 7 Cattedra Unesco “Educazione alla Salute e allo Sviluppo Sostenibile”, University of Naples Federico II, 80131 Naples, Italy; 8 Department of Biomedical Sciences, University of Padova, 35131 Padova, Italy; antonio.paoli@unipd.it; 9 Research Center for High Performance Sport, UCAM Catholic University of Murcia, 30107 Murcia, Spain

**Keywords:** sport nutrition, oxidative stress, Mediterranean diet, polyphenols, muscle

## Abstract

Several antioxidants play an important role in improving athletic performance. Increased inflammation and oxidative stress during physical performance result in the production of free radicals, including reactive oxygen species (ROS), which leads to fatigue, muscle damage, and impaired performance. However, moderate and transient increases in ROS are physiologically essential, acting as signaling mediators that trigger adaptive cellular responses. Despite their harmful effects on athletic performance, ROS may also enhance physical protection by acting as signaling molecules against increased physical stress. Healthy dietary patterns such as the Mediterranean diet (MD) may contribute to decrease oxidative stress thanks to its content in fruits, vegetables, olive oil, legumes, and herbs/spices. Indeed, the beneficial effects of the MD can be attributed not only to its antioxidant properties but also to its well-documented anti-inflammatory effects, lipid-modulating actions, immune-supportive functions, and modulation of gut microbiota composition, which collectively influence metabolic and physiological resilience. The MD also plays a key role in competitive sport and training. In addition, several researchers have reported that the MD is essential for reducing risk of injury and illness, recovering and adapting between bouts of activity, and enhancing performance. In this context, following the key principles of an MD could also represent a useful framework for good dietary in competitive athletes. In this narrative review, we discuss the potential effects of antioxidants in sport and the impact of individual foods or compounds of the MD on oxidative stress and exercise performance in competitive athletes and physically active individuals. The potential modifications which could be made to the MD will be highlighted to maximize health and performance effects, in accordance with contemporary sports nutrition practices.

## 1. Introduction

Physical exercise is a powerful physiological stimulus that enhances metabolic efficiency, cardiovascular fitness, and neuromuscular function. However, the metabolic acceleration required to sustain muscle contraction inevitably increases the production of reactive oxygen species (ROS), primarily through mitochondrial electron leakage, NADPH oxidase activation (particularly NOX2 at the sarcolemma), and xanthine oxidase activity [1]. Rather than being purely detrimental, ROS also function as essential signaling mediators. At controlled levels, they activate redox-sensitive transcription factors and kinases, such as nuclear factor erythroid 2-related factor 2 (Nrf2), mitogen-activated protein kinase (MAPK), and peroxisome proliferator-activated receptor gamma coactivator 1-alpha (PGC-1α), promoting mitochondrial biogenesis, angiogenesis, and antioxidant enzyme expression [2,3]. In this way, ROS serve as critical triggers for exercise-induced adaptations [4], occurring within specific intensity-dependent redox thresholds. Yet, this adaptive potential depends on a delicate redox balance. When ROS production increases beyond the buffering capacity of endogenous antioxidant systems (i.e., as consequence of high exercise intensity or duration), redox-sensitive signaling shifts from an adaptive to a damaging profile, impairing force generation and accelerating inflammatory and oxidative injury [5]. Chronic or repeated episodes of unresolved oxidative stress may contribute to fatigue, immune dysregulation, and reduced training capacity. Thus, optimizing redox homeostasis—rather than eliminating ROS—has become a central objective in sports nutrition and exercise physiology. Consistent with a hormetic framework, exercise-induced ROS act as essential signaling molecules for performance and adaptation, whereas both excessive oxidative burden and non-selective suppression of redox signaling can impair muscle function, inflammatory remodeling, and the capacity to adapt to repeated training stimuli [4,6].

Dietary patterns play a fundamental role in shaping systemic and cellular redox environments. Beyond macronutrient composition, bioactive phytochemicals may influence mitochondrial function, inflammatory tone, and the efficiency of exercise recovery [4]. In this context, the Mediterranean Diet (MD) has emerged as one of several antioxidant-rich dietary patterns—alongside the DASH and Nordic diets. It is uniquely characterized by its high monounsaturated-fat content, extra virgin olive oil (EVOO)-derived phenolics, and long-term evidence base in cardiometabolic outcomes, making it particularly relevant to redox physiology. More than a regional dietary tradition, the MD is a biochemically coherent antioxidant system, characterized by high consumption of fruits, vegetables, legumes, nuts, minimally processed whole grains, fish, and particularly EVOO, alongside moderate intake of wine and low consumption of refined sugars and saturated fats [7]. The MD’s antioxidative and anti-inflammatory potential stems from the synergistic interactions among its key bioactive compounds [8]. As an example, polyphenols from EVOO, such as hydroxytyrosol and oleuropein, have been shown to modulate Nrf2, enhancing transcription of endogenous antioxidant enzymes including superoxide dismutase, catalase, and glutathione peroxidase [9,10]. Concurrently, these polyphenols can downregulate specific pathways playing a role as central mediators of inflammation, thereby reducing cytokine expression and pro-oxidant compound levels [11,12,13,14,15], thus contributing in promotion of a more favorable immune environment during recovery.

Importantly, the MD delivers antioxidants within its peculiar dietary features, in terms of typical Mediterranean foods rather than as isolated supplemental compounds. This distinction has significant physiological implications. Although direct interventional studies investigating MD-mediated redox effects in athletic populations remain limited, evidence from non-athletic and mixed cohorts supports the hypothesis that, in athletes, MD may promote a balanced and adaptive modulation of redox homeostasis rather than indiscriminate ROS suppression. This effect is postulated to occur through the combined action of multiple bioactive compounds that can activate redox-sensitive pathways involved in cellular adaptation, including Nrf2-dependent upregulation of endogenous antioxidant defenses, preservation of mitochondrial function and biogenesis, and modulation of mitochondrial respiratory efficiency and cyclooxygenase-related signaling. Unlike high-dose single-antioxidant supplementation, which may blunt exercise-induced adaptations, the MD provides a diverse antioxidant network capable of attenuating excessive exercise-induced oxidative stress while preserving physiological ROS signaling required for performance and training adaptation; however, in athletes these mechanisms are currently inferred from evidence obtained in other experimental settings and warrant direct investigation [16].

Clinical studies reinforce the MD’s capacity to modulate oxidative stress and support metabolic resilience. Higher adherence to the MD is associated with reduced lipid peroxidation and lower DNA oxidative damage in observational and interventional studies, although causality cannot be inferred due to potential confounding [17,18,19]. These outcomes are directly relevant to physically active populations, who routinely experience substantial oxidative and inflammatory challenges. By improving basal redox status, the MD may support more rapid recovery between training sessions, sustain immune competence during heavy training periods, and promote long-term adaptation capacity.

Emerging evidence in athletic contexts aligns with these findings. Athletes consuming diets richer in natural antioxidants frequently display lower biomarkers of exercise-induced muscle damage (i.e., DOMS, malondialdehyde, creatine kinase, muscle pain and soreness), reduced inflammatory cytokine profiles (i.e., C-reactive protein, interleukins), improved vascular responsiveness, and enhanced subjective recovery [4]. However, these associations are largely derived from observational or short-term intervention studies, and causality cannot be firmly established. Moreover, consistent dietary polyphenol exposure may facilitate adaptive mitochondrial remodeling and improve oxygen utilization efficiency—key determinants of performance in endurance and intermittent high-intensity sports [4]. Nonetheless, the magnitude and consistency of these effects remain variable across studies, suggesting that individual training status, dietary background, and study design may influence outcomes. Importantly, these benefits appear to complement rather than impair the cellular signaling required for training adaptation [4].

Yet, despite growing interest, application of the MD within sports nutrition remains relatively underdeveloped. Most research on the MD has focused on chronic disease prevention, leaving open questions regarding optimal integration strategies for athletic training cycles, energy periodization, and sport-specific metabolic demands. Individual variability in redox responses further suggests that personalized modulation of MD components—particularly in relation to training load and recovery state—may be necessary [16], particularly regarding interindividual variability in polyphenol metabolism (gut microbiota-dependent), omega-3 fatty acid incorporation into membranes, and responses to EVOO phenolics.

The aim of this narrative review is therefore to synthesize current evidence on exercise-induced oxidative stress and the physiological roles of dietary antioxidants, with a specific focus on the MD as a comprehensive and adaptive antioxidant system. We identify current gaps—including the scarcity of longitudinal trials in athletes, inconsistent biomarker standardization, and limited integration of dietary patterns into sport-specific nutrition periodization. Although several mechanisms underlying antioxidant activity are shared between athletes and physically active individuals, the primary focus of this review is on competitive and high-performance athletes, for whom oxidative stress dynamics, recovery demands, and dietary requirements differ substantially from those of recreational exercisers. Also, we critically examine the molecular mechanisms through which MD constituents influence redox regulation, recovery, and training adaptation, and evaluate the potential of MD-based nutrition to enhance performance and long-term athlete health. Given the narrative nature of the manuscript, the literature included was identified through a non-systematic search performed in PubMed and Scopus up to September 2025, using combinations of keywords related to antioxidants, oxidative stress, Mediterranean diet, and sport performance. Priority was given to human studies and recent reviews, while additional references were selected through forward and backward citation tracking. This approach aims to provide a comprehensive and integrative synthesis rather than an exhaustive systematic analysis.

## 2. Antioxidants and Sports Performance

Regular physical activity confers broad systemic benefits, including improved metabolic, cardiovascular, and neurocognitive function. However, strenuous or unaccustomed exercise can induce muscle damage, inflammation, and oxidative stress due to increased production of ROS. During muscular contraction, ROS arise primarily from mitochondrial oxidative phosphorylation, xanthine oxidase (XO) activity during purine metabolism, and NADPH oxidases (NOX/DUOX), which generate superoxide (O•−2), hydrogen peroxide (H_2_O_2_), and hydroxyl radical (•OH) [1]. Additional contributors include catecholamine auto-oxidation and uncoupled mitochondrial respiration [20,21,22]. While physiological levels of ROS generated within skeletal muscle fibers support excitation—contraction coupling and muscular force production, excessive or poorly regulated ROS accumulation—arising from both intramuscular and non-mitochondrial sources such as NADPH oxidases and xanthine oxidase–may impair performance, promote contractile failure, and contribute to tissue injury [5,6]. Controlled, compartmentalized increases in ROS are also central to redox signaling and exercise adaptation, driving the upregulation of endogenous antioxidant defenses and redox-sensitive signaling pathways [4,6]. Accordingly, low oxidant concentrations facilitate muscle contraction, whereas non-selective exogenous antioxidant administration can paradoxically reduce contractile function, an effect reversible by adding H_2_O_2_ [5]. Importantly, sustained supplementation with high doses of exogenous antioxidants has been reported to dampen physiological redox signaling during exercise, thereby blunting key training-induced adaptations such as mitochondrial biogenesis, activation of endogenous antioxidant systems, improvements in insulin sensitivity, and structural remodeling of skeletal muscle, particularly under endurance and high-intensity training conditions [6].

NOX4 may act as a metabolic sensor linking ATP turnover to ROS signaling via its ATP-binding regulatory motif, and evidence suggests NOX enzymes can modulate calcium release through ryanodine receptor type 1, supporting contractile performance [23,24]. XO-derived superoxide further contributes to force production during isometric exercise [25]. Trained individuals demonstrate greater tolerance to exercise-induced oxidative stress than untrained individuals, in part due to enhanced antioxidant enzyme expression and redox buffering capacity [4]. Key adaptive signaling pathways include Nrf2-mediated transcription of antioxidant systems [2] and PGC-1α-driven mitochondrial biogenesis, regulated via MAPK and nuclear factor kappa-light-chain-enhancer of activated B cells (NF-κB) signaling [3]. Exercise modality modulates these responses: aerobic exercise generally enhances antioxidant enzyme activities such as catalase, glutathione peroxidase (GPx), and superoxide dismutase (SOD), promoting more efficient ROS neutralization, whereas resistance training increases structural and neuromuscular adaptations but can initially elevate oxidative and inflammatory stress [4,26]. High-intensity exercise and sprinting rely primarily on anaerobic metabolism and hypoxanthine accumulation, activating XO and NOX to produce ROS [27]. These ROS bursts may mediate hypertrophic signaling through pathways such as phosphatidylinositide 3-kinase/protein kinase B (PI3K/Akt), influenced by redox-sensitive regulators [28]. Notably, environmental conditions such as high altitude further exacerbate oxidative stress due to hypobaric hypoxia, leading to increased ROS production, impaired antioxidant defenses, and potential mitochondrial and DNA damage [29,30]. Reduced Mn-SOD expression and elevated Ku70 in high-altitude exposure underscore the enhanced oxidative challenge to muscle tissue [31]. Although antioxidant supplementation has been proposed to counteract altitude- or exercise-associated oxidative stress, inconsistent findings highlight the need for individualized strategies that avoid impairing beneficial redox signaling [32].

Because exercise-induced ROS production can be either beneficial or detrimental depending on concentration, duration, and training status, strategies that fine-tune redox homeostasis are essential for optimizing performance. Therefore, attention has turned to antioxidant compounds—particularly those derived from diet—as potential modulators of these redox-sensitive pathways.

The increasing interest in antioxidant supplementation in athletic contexts stems from its proposed potential to mitigate muscle damage, limit exercise-induced oxidative stress, improve performance, and reduce longer-term health risks associated with repeated high-intensity training [4]. Optimal antioxidant status can be achieved through a balanced diet rich in fruits, vegetables, legumes, and dietary fiber, which provides vitamins and minerals in physiologically synergistic ratios that enhance antioxidant effectiveness [4,27,33]. Quercetin, in particular, has been investigated for its effects on exercise performance. Supplementation for seven days has been shown to modestly increase VO_2_max and significantly extend time to fatigue in untrained but healthy individuals, suggesting meaningful improvements in endurance capacity that may be relevant not only for athletic or military settings but also for general health promotion [34].

Diets naturally high in plant-derived antioxidants have been consistently associated with reduced systemic inflammation. To evaluate their effects on exercise-induced oxidative stress exacerbated by environmental stressors such as altitude, a study was conducted on elite endurance athletes consuming antioxidant-rich foods during a three-week training camp at 2320 m. Athletes showed increased antioxidant capacity and partially attenuated systemic inflammation, although oxidative stress markers and acute cytokine responses remained unchanged [35]. However, other trials, such as those assessing flavonoid-rich juice intake in elite swimmers, showed no significant improvements in inflammation, oxidative stress, immunity, or metabolic status beyond enhanced nutrient intake [36].

Studies evaluating specific antioxidant supplements have yielded variable results. Watermelon intake increased circulating L-citrulline and L-arginine, improved antioxidant capacity, and supported exercise energy demands, but did not significantly influence inflammation or immune parameters [37]. Supplementation with natural pomegranate juice reduced acute and delayed muscle soreness and inflammatory markers, and improved recovery and training performance in elite weightlifters [38]. Conversely, co-supplementation with vitamins C and E during intermittent running did not reduce oxidative stress, inflammation, or improve muscle recovery [39]. During a 50 km ultramarathon, antioxidant vitamin supplementation reduced lipid peroxidation but did not prevent inflammation [40], and in kayakers, a four-week antioxidant regimen did not protect against oxidative or inflammatory damage and may have impaired recovery [41].

Antioxidants play also a recognized role in recovery following strenuous exercise, as elevated ROS contributes to muscle damage, DOMS, and temporary performance reductions [4,42,43]. However, results from interventions remain inconsistent. Pomegranate juice improved oxidative stress recovery in strength training [38]. Melatonin supplementation demonstrated protective effects on skeletal muscle and improved glutathione peroxidase activity [44,45]. L-carnitine improved perceived recovery and reduced creatine kinase (CK) levels [46]. Vitamin E and C supplementation reduced markers of lipid peroxidation and oxidative stress in some contexts [47], but not muscle soreness or CK levels following eccentric or plyometric exercise [48,49]. Evidence generally conclude that antioxidant supplementation has limited consistent benefit for reducing DOMS [50,51,52], likely due to differences in exercise modality, participant characteristics, dosage, and supplementation duration.

Research on performance outcomes has also produced mixed findings. Quercetin supplementation improved performance in badminton players and cyclists [53,54], but showed no benefit in swimmers or untrained subjects [55,56]. Limited data on resveratrol suggest inconsistent effects on performance adaptations [57]. Nitrate-rich beetroot juice has shown improved exercise efficiency and performance in several studies [58,59,60], though its benefits may be less pronounced in highly trained athletes [61]. Taken together, these findings indicate that performance improvements are not uniform across populations or exercise modalities, and that responses to antioxidant-rich supplements are influenced by training status, baseline diet, and study design. Therefore, current evidence does not allow firm conclusions regarding systematic ergogenic effects.

**Figure 1:**
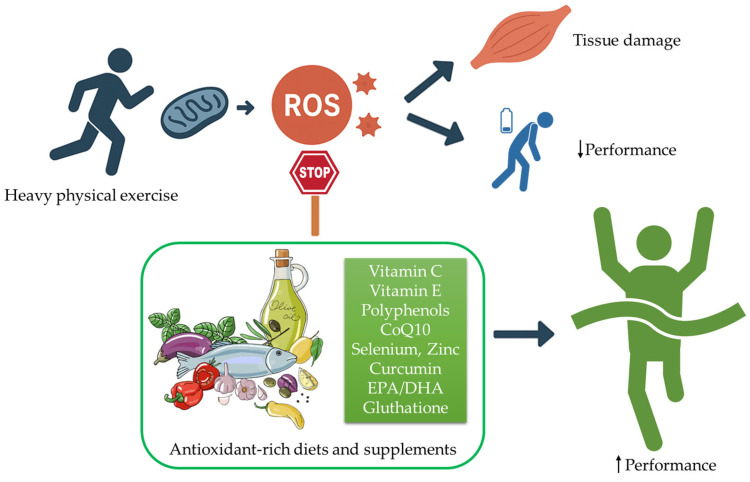
**Interaction Between Exercise-Induced Oxidative Stress and Antioxidant Support in Sports Performance**. Intense physical exercise increases the production of reactive oxygen species (ROS), exceeding the capacity of endogenous antioxidant defenses and contributing to oxidative damage in skeletal muscle tissue. This imbalance can impair recovery dynamics and compromise athletic performance. Dietary patterns rich in naturally occurring antioxidants, as well as targeted antioxidant supplementation when appropriately applied, may attenuate exercise-induced oxidative stress and support performance outcomes. These recommendations were derived and adapted from Clemente-Suárez et al., 2023 [4].

Given the complexity of exercise-induced oxidative stress and the variability in individual training demands, athletes and practitioners frequently consider antioxidant supplementation as a strategy to support performance and recovery. However, evidence remains mixed, and supplements differ substantially in their effects, mechanisms, and appropriate use. Below, we provide an overview of the main antioxidant compounds for which effects in athletes have been most consistently studied, outlining practical considerations related to dose, timing, and duration, while also cautioning where uncertainties remain (Figure 1).

As previously reviewed [4], vitamin C is one of the most commonly used antioxidants in sport. It contributes to neutralizing ROS and modulating inflammation after high-intensity exercise. Daily intake typically ranges from 500 to 2000 mg [4], though doses up to 3 g/day have been applied in prolonged endurance efforts [62]. Importantly, the timing of intake may influence outcomes: pre-exercise supplementation has been associated with reduced muscle damage and inflammation [63], while post-exercise intake may support recovery and reduce soreness [48]. Most protocols extend over 4–6 weeks leading up to competition [64], although care must be taken to avoid excessive, long-term dosing, which may interfere with redox-mediated adaptation [4].

Vitamin E has similarly been studied for its role in protecting cell membranes from oxidative damage. Doses of 400–800 IU, typically administered either acutely before exercise or in short loading phases, have been shown to reduce markers of muscle oxidation [65,66]. However, chronic intakes exceeding 1000 IU/day should be avoided due to risks including impaired coagulation. In general, vitamin E appears most appropriate for short-term, targeted use, rather than continuous supplementation [4].

Among polyphenols, resveratrol has attracted interest due to its anti-inflammatory and antioxidant properties. Effective supplementation in exercise studies ranges from 250–500 mg/day over 4–8 weeks [67,68]. While some data suggest improvements in metabolic efficiency and recovery processes, evidence in trained athletes remains limited, and more research is needed to clarify performance-specific benefits [4].

Coenzyme Q10 (CoQ10) plays a key role in mitochondrial electron transport and cellular energy metabolism. Supplementation at 100–300 mg/day for 4–12 weeks has been associated with reduced muscle damage and improved performance measures in both trained and untrained individuals [69,70]. CoQ10 appears to be beneficial when incorporated into both pre-competition preparation and recovery phases, particularly in sports requiring sustained metabolic output [4].

Selenium, as part of the glutathione peroxidase antioxidant enzyme system, contributes to redox balance and immune function. Doses of 200–400 µg/day have shown improvements in aerobic performance and muscle strength, though intakes must not exceed 400 µg/day due to toxicity concerns [71,72]. Supplementation protocols generally range 4–12 weeks [4].

Curcumin, found in turmeric, has been widely studied for reducing inflammation and soreness. Doses of 400–500 mg twice daily have been effective in improving recovery, while 90–500 mg/day may enhance performance [4,73,74,75,76]. Both pre- and post-exercise supplementation are supported, depending on whether the objective is prevention or recovery [4].

Omega-3 fatty acids (EPA + DHA) play a central role in moderating exercise-induced inflammation and oxidative stress. Intakes of 1–5 g/day, particularly around 3 g/day, sustained for 4–12 weeks, have demonstrated improvements in recovery and performance in endurance contexts [77,78]. Supplementation can be strategically integrated into both the training and competition phases [4].

Zinc contributes to antioxidant enzyme function and immune health. Doses of 15–30 mg/day for at least 4–6 weeks may enhance antioxidant status [79,80], though higher intakes risk immune suppression and impaired nutrient absorption [81].

Finally, glutathione and its precursor N-acetylcysteine (NAC) have been evaluated for restoring endogenous antioxidant defenses. Oral glutathione at 450–1000 mg/day over 3 weeks has shown reductions in oxidative stress markers [82,83], though effects on performance remain inconsistent. NAC doses vary widely (1.2–20 g/day) and can produce adverse effects, particularly at higher doses [84].

Collectively, findings indicate that while antioxidants influence exercise-associated oxidative stress and recovery, supplementation effects are inconsistent, and whole-food antioxidant intake may offer a more effective and physiologically balanced approach.

## 3. Mediterranean Diet

The MD is widely recognized as a comprehensive lifestyle model integrating nutritional choices with cultural, social, economic, and environmental dimensions [85]. Originating from the longstanding interaction between food resources and the traditional eating habits of populations in the Mediterranean basin, the MD emphasizes seasonality, biodiversity, and the preferential consumption of fresh, locally sourced foods [7,86]. As acknowledged by UNESCO, the MD constitutes not only a dietary pattern but a cultural heritage that reflects sustainable agricultural practices and their interrelationship with community life [7]. Traditionally associated with regions including Greece, southern Italy, and other areas of southern Europe, the MD displays local variations but shares several core nutritional principles [87,88]. These include the daily consumption of whole grains, fruits, vegetables, nuts, and low-fat dairy products; the preferential use of olive oil (OO) as the main lipid source; moderate intake of red wine with meals; moderate consumption of fish, poultry, eggs, potatoes, and sweets; limited red meat intake; and regular physical activity [88,89].

The MD is consistently associated with longevity, disease prevention, and enhanced quality of life, primarily attributed to its content of bioactive compounds with antioxidant and anti-inflammatory properties [90,91]. Main interrelated pathways include lipid-lowering effects, anti-oxidative and anti-inflammatory actions, hormonal and gut microbiota modulation, and inhibition of nutrient-sensing pathways [92]. Contemporary research increasingly emphasizes the synergy among dietary components and the importance of considering culinary practices, food production, and sociocultural behaviors when evaluating the MD as a holistic lifestyle system [7,93].

A key component of the MD relates to the bioactive molecules present in its primary food sources [85]. Marine omega-3 fatty acids from fish and seafood (particularly EPA and DHA) exert cardioprotective effects and have been associated with reduced coronary heart disease risk in high-risk populations, as supported by meta-analytic evidence from randomized controlled trials [94,95]. Main sources of polyunsaturated fatty acids are also represented by nuts, which have been linked to improved cardiovascular and metabolic outcomes [88,96,97,98]. Similarly, OO, particularly EVOO, constitutes the principal lipid source in the MD, rich, among the other bioactive compounds, in monounsaturated fatty acids [99]. Evidence from observational studies and controlled research indicates that OO consumption decreases chronic disease risk and improves metabolic and inflammatory biomarkers [100,101], including reductions in markers of oxidative DNA damage and pro-inflammatory cytokines [102,103].

The MD supports and modulate the body’s antioxidant network against pro-oxidant species, in particular reactive oxygen nitrogen species [17,104]. Its pronounced antioxidant potential is attributable to the high content and synergistic interplay of bioactive compounds, mainly polyphenols [105]. Polyphenols, secondary metabolites in plants, are chemically defined by phenolic rings with hydroxyl groups and are categorized into flavonoids, phenolic acids, polyphenolic amides, and additional subclasses such as stilbenes and lignans. These categories reflect distinct biochemical properties and contribute differentially to the physiological effects associated with polyphenol-rich dietary patterns [106].

Polyphenols regulate ROS and immune responses via modulation of signaling pathways such as NF-κB, MAPK, and PI3K/Akt, enhancing endogenous antioxidant enzymes including SOD, catalase, GPx, and GPR40 [11,12,13,14]. They also exert genomic and epigenomic effects, modulating transcriptional programs related to NF-κB, MAPK, and Nrf2 [15].

Polyphenols are present across a broad range of fruits, vegetables, and plant-derived beverages such as red wine and tea, with concentrations varying among foods [107]. Some polyphenols are characteristic of specific foods, such flavanones in citrus fruits, whereas others, like quercetin, are widely distributed. Final polyphenol content is influenced by climate and culinary processes [108]. In this sense, due to the peculiar pedoclimatic characteristics of Mediterranean areas and typical features related to food choices and culinary instructions for their preparation, the MD represents a dietary pattern that provides a high intake of polyphenols. Typical Mediterranean foods, indeed, are rich sources of polyphenols, including EVOO, presenting oleuropein, tyrosol, hydroxytyrosol, and secoiridoids, that significantly contribute to its anti-inflammatory and antioxidant effects [9,10]. Legumes, whole grains, and nuts further reinforce the dietary pattern. These foods supply fiber, vitamins, minerals, and phenolic compounds [85,109]. Fruit and vegetable intake represents another central pillar of the MD. These foods provide essential micronutrients and a wide array of polyphenols, mainly flavonoids, which contribute to antioxidant defense mechanisms [89,110]. Consistent data show that higher intake of fruits and vegetables is associated with reduced all-cause mortality, cardiovascular diseases, type 2 diabetes, cancer, and obesity risk [111,112,113,114,115,116]. In addition to foods, typical Mediterranean spices and herbs are valid sources of polyphenols [117]. Moreover, plant-derived beverages are also included in the MD. In particular, moderate red wine consumption is permitted, providing resveratrol, a polyphenol associated with protective effects against chronic diseases and metabolic syndrome features [88,118].

The abundance of polyphenols in several Mediterranean foods, thus, highlights the antioxidant potential of this dietary pattern [90]. Large epidemiological studies, such as the ATTICA study, have already established a positive correlation between greater adherence to the MD and elevated total antioxidant capacity levels in healthy adults [119]. More compelling evidence comes from the PREDIMED study. In a subsample of the PREDIMED trial, participants randomized to the MD supplemented with virgin olive oil (VOO) or the MD supplemented with nuts showed significant increases in plasma non-enzymatic antioxidant capacity (NEAC) after one year [17]. These findings confirmed that adherence to the MD pattern, particularly when enriched with key components like VOO or nuts, effectively enhances systemic antioxidant defenses in high-risk subjects. Furthermore, a 3-year follow-up within the PREDIMED cohort showed that an MD rich in VOO was associated with high plasma antioxidant capacity [18]. Recently, the historically recognized relationship between adherence to the MD and reduced oxidative stress has been further pointed out by a study reporting the role of this dietary pattern in slowing the biological aging. Mediation analyses indicate that these benefits are, at least in part, driven by the diet’s capacity to reduce inflammation and enhance antioxidant defense mechanisms [19]. Similarly, a recent meta-analysis examining adherence to the MD demonstrated improvements in oxidative stress biomarkers in the intervention groups compared to control diets, although most effects did not reach statistical significance. Notably, reductions in malondialdehyde (MDA) and 8-hydroxy-2′-deoxyguanosine (8OHdG) were observed among individuals following the MD. According to the authors, the lack in statistical significance may be due to substantial heterogeneity across studies, reflecting differences in genetic background, lifestyle behaviors, degree of dietary adherence, and variability in the control diet conditions. However, they focus on the importance to recognize that even modest shifts in oxidative biomarkers may hold clinical relevance over time, particularly in the context of chronic disease prevention and metabolic health trajectories [120]. This complex framework linking the unique characteristics of Mediterranean foods with various health benefits, mainly mediated by their antioxidant effect, highlights the nutraceutical potential of the MD as an added value beyond its mere nutritional characteristics [8].

The antioxidant potential of MD exhibits complexity, suggesting the existence of crucial tuning mechanisms within the plasma antioxidant network aimed at maintaining a dynamic physiological homeostasis. Analysis of the PREDIMED data revealed that the effectiveness of antioxidant supplementation through diet is highly related to the baseline NEAC status of the participants. Specifically, participants who were in the lowest quartile of plasma ferric reducing antioxidant potential at baseline demonstrated a significant increase in their NEAC levels regardless of the intervention (MD + VOO, MD + nuts, or even the control low-fat diet). Conversely, subjects in the highest baseline NEAC quartile tended to show a decrease in their plasma antioxidant levels [17]. This mechanism of tuning suggests that the organism regulates its antioxidant status to prevent redox overloading, thereby maintaining physiological homeostasis [121,122]. This observation has profound implications, contrasting with the often-negative outcomes observed in meta-analyses concerning high-dosage galenic antioxidant supplementation on overall mortality and cardiovascular diseases [123]. The evidence suggests that increasing antioxidant intake through dietary patterns is most beneficial when the body requires it, such as in states of low baseline antioxidant capacity or existing oxidative stress conditions such as during exercise as supported by the hormesis theory [124,125].

Overall, the MD exerts comprehensive antioxidant action primarily through the concerted activity of its polyphenol-rich foods and other bioactive constituents, which collectively modulate redox-sensitive transcriptional networks, upregulate endogenous antioxidant defenses, and attenuate oxidative and inflammatory cascades. This integrated molecular interplay ultimately contributes to the preservation of redox homeostasis, the mitigation of cellular damage, and the promotion of long-term cardiometabolic and systemic health.

## 4. Antioxidants of the Mediterranean Diet and Physical Performance in Competitive Athletes

Appropriate nutrition plays is fundamental for supporting training and competition, sustaining performance, reducing of injury and illness risk, promoting recovery, and optimizing long-term health [126]. Among dietary strategies, the MD has gained attention due to its intrinsic nutritional profile and health-promoting properties [7].

A central feature of the MD for athletes is its carbohydrate content, naturally provided by whole grains, legumes, and fruit, supporting energy availability, performance, and recovery [127,128,129].

The MD has also been recognized as a healthy, palatable, and cost-effective dietary pattern that provides a rich nutrient profile with well-documented benefits for cardiovascular, metabolic, and cognitive health [7], and recent literature suggests that it may also serve as a performance-enhancing and health-promoting nutritional model in competitive sporting populations [130]. One defining feature is its high content of antioxidant and anti-inflammatory bioactive compounds. Moderate-to-high intake of fruits and vegetables (3–5 portions/day) ensures a substantial supply of vitamins C and E, carotenoids, flavonoids, and polyphenols, contributing to redox balance [131].

Adequate antioxidant intake supports recovery and performance by mitigating exercise-induced oxidative stress. The MD provides a comprehensive array of nutrients and phytochemicals acting synergistically to maintain redox homeostasis, offering a theoretical basis for optimizing adaptation and performance. This hypothesis has been explored in recent human studies (detailed in Table 1), including a systematic review and meta-analysis by Fiorini and colleagues [130], which aimed to determine whether the MD could serve as a reference dietary pattern for athletes. Following the PRISMA guidelines and registered under PROSPERO (CRD42023459039), this review analyzed nine studies including 192 healthy competitive or elite adult athletes (mostly males) performing at least six hours of training per week. Five of these studies employed a cross-sectional design, with sample sizes ranging from 10 to 43 athletes practicing different sports disciplines. Among them, five studies reported an impact of MD adherence on athletic performance, and four described a positive association. Specifically, greater adherence to the MD correlated with higher aerobic and anaerobic power, greater explosive strength, and, indirectly, with lower body fat percentage, while adherence levels varied from low to high. Despite these qualitative findings, the quantitative meta-analysis revealed no significant overall effect of MD adherence on performance outcomes [standardized mean difference (SMD): 0.00; 95% CI: −0.26 to 0.25], likely reflecting the small number of studies, methodological heterogeneity, and low-to-moderate quality of evidence. Nonetheless, the authors concluded that the available data suggest a general positive influence of the MD on athletic performance, supporting its role as a beneficial and health-promoting dietary model for athletes. These findings are consistent with mechanistic evidence linking the MD to enhanced mitochondrial function, improved endothelial health, reduced oxidative stress, and more favorable lipid and inflammatory profiles—all factors that contribute to endurance, strength, and recovery.

The MD’s potential to improve cardiovascular efficiency, optimize lipid metabolism through a higher intake of monounsaturated and polyunsaturated fatty acids, and maintain an optimal inflammatory balance may collectively sustain high training loads while reducing the physiological burden of oxidative damage [132]. Moreover, the diet’s high content of dietary fiber and polyphenols can beneficially modulate the gut microbiota, promoting bacterial species that support immune function and nutrient absorption [133], thus contributing indirectly to athletic resilience and performance.

In addition to performance-related outcomes, no current studies have directly demonstrated that adherence to the MD improves fatigue outcomes in competitive athletes. However, given that oxidative stress is implicated in the development of exercise-induced fatigue, and that several MD components exhibit antioxidant and anti-inflammatory properties, it is biologically plausible to hypothesize that high adherence to the MD may also contribute to the modulation of fatigue-related processes. This hypothesis remains speculative, and further athlete-specific research is required to confirm these effects and clarify the underlying mechanisms.

In summary, the MD emerges as a promising and physiologically coherent dietary strategy for competitive athletes, characterized by its richness in antioxidants, anti-inflammatory agents, and high-quality macronutrients. Although quantitative evidence remains limited, converging lines of mechanistic, observational, and interventional research indicate that adherence to the MD is associated with improved recovery, reduced oxidative and inflammatory stress, better body composition, and possibly enhanced aerobic and anaerobic performance. Thus, beyond its well-established health benefits in the general population, the MD may represent a sustainable and evidence-based model for optimizing performance, recovery, and long-term well-being in athletes across different disciplines.

**Table 1:** Summary of studies and reviews on the Mediterranean diet, antioxidant mechanisms, and athletic performance.

Study	Population	Design	Main Findings	Outcome on Performance
Fiorini et al., 2025 [130]	192 competitive/elite athletes (various sports)	Systematic review & meta-analysis (PROSPERO CRD42023459039)	MD adherence positively related to aerobic/anaerobic power, explosive strength, and lower body fat %—no pooled effect on performance (SMD 0.00; CI −0.26 to 0.25)	General positive trend; inconclusive quantitative effect
Kozjek et al., 2025 [126]	Athletes	Narrative review	Nutrition optimizes immune function, recovery, and reduces illness/injury risk in athletes	Rationale for adopting high-quality dietary patterns such as MD
Guasch-Ferré & Willett, 2021 [7]	General population	Narrative review	MD described as healthy, palatable, and cost-effective; rich in micronutrients	Establishes MD as a model dietary pattern
Griffiths et al., 2022 [16]	General population	Review	Defines key MD components and rationale for optimizing health	Provides mechanistic basis for MD-health links
Mazzocchi et al., 2019 [134]	General population	Review	Olive oil and MD phenolics linked with improved vascular, metabolic, and immune function	Indirect benefits on endurance and oxidative balance
Arpón et al., 2016 [135]	Adults adhering to MD	Observational epigenetic study	MD alters methylation of inflammation-related genes	Epigenetic anti-inflammatory mechanism
Perez-Martinez et al., 2006 [136]	Sixteen healthy men followed three 4-week diets: Western diet, MD and low-fat diet enriched in alpha-linolenic acid	RCT	Western diet increased 2.7-fold NF-κB compared with the Mediterranean diet (*p* = 0.038) and 1.79-fold with the alpha-linolenic acid diet (*p* = 0.07). No differences were found between the last two. Furthermore, an increase in plasma VCAM-1 was observed with the Western diet (*p* < 0.05)	Confirms systemic anti-inflammatory effect
Calder, 2013 [137]	Human & clinical	Review	n-3 PUFAs reduce inflammation, support immune modulation and muscle recovery	Improves recovery and reduces exercise-induced inflammation
McAnulty et al., 2011 [138]	Running athletes	RCT	Blueberry supplementation during prolonged running enhanced NK cell counts and reduced oxidative stress	Antioxidant support; improved recovery markers
Howatson et al., 2010 [139]	Recreational Marathon runners	RCT	Cherry juice reduced post-marathon inflammation and accelerated recovery	Improved recovery kinetics

Abbreviations: Mediterranean diet, MD; standardized mean difference, SMD; Nuclear factor kappa-light-chain-enhancer of activated B cells, NF-κB; Vascular cell adhesion protein 1, VCAM-1; polyunsaturated fatty acids, PUFAs; natural killer, NK; randomized controlled trial, RCT.

## 5. Antioxidants of the Mediterranean Diet and Sports: Nutrition Recommendations

Given the recognized health and performance-related benefits of the MD, evaluating adherence to this dietary pattern among athletes is an important step in determining whether sport-specific MD interventions may be warranted. Evidence indicates that compliance with established sports nutrition guidelines is frequently suboptimal, particularly with respect to carbohydrate intake during training and competition, where many athletes fail to achieve recommended levels [140,141]. While macronutrient periodization remains essential and must be adapted to the unique demands of each sport and training phase, attention has increasingly turned toward optimizing the intake of bioactive dietary constituents—particularly those with antioxidant and anti-inflammatory properties—that are characteristic of the MD, as summarized in Table 2 according to the nutritional recommendations provided by Griffiths et al., 2022 [16].

Position statements from leading authorities including the American College of Sport Medicine [142] and International Society of Sports Nutrition [143] emphasize the importance of diets rich in antioxidants, especially from fruits and vegetables, for athletes experiencing repeated bouts of exercise-induced oxidative stress. This recommendation is supported by evidence demonstrating that diets naturally high in antioxidants may attenuate exercise-induced increases in oxidative damage. Watson et al. [144] reported that trained athletes consuming a habitual antioxidant-rich diet exhibited lower ratings of perceived exertion during exercise and reduced post-exercise F(2)-isoprostanes, a robust index of lipid peroxidation, compared with when antioxidant-rich foods were restricted. The authors suggested that individuals engaged in frequent high-intensity exercise likely require greater exposure to dietary antioxidant compounds than sedentary populations, and that these demands can generally be met through a nutrient-rich diet without routine reliance on supplementation [144]. The MD aligns with these recommendations via moderate-to-high fruit and vegetable intake, regular inclusion of EVOO, and modest red wine consumption, all providing polyphenols with antioxidant activity. OO phenolics, such as hydroxytyrosol and oleuropein, mitigate oxidative stress and enhance mitochondrial function [145,146]. In an exercise context, Musumeci et al. [147] demonstrated that rats consuming an EVOO-enriched diet exhibited reduced markers of oxidative injury (e.g., hydroperoxides and thiobarbituric acid-reactive substances) and increased antioxidant defenses (e.g., NEAC and Hsp70 expression) following exhaustive exercise relative to standard chow-fed controls. In human studies, consumption of wine (approximately 240 mL/day) and the MD as a whole have been associated with reduced oxidative DNA damage, including lower levels of oxidized guanine derivatives such as 8-OHdG in peripheral leukocytes [148]. Additional trials show increased plasma antioxidant capacity with 300–400 mL/day of wine for two weeks [149,150,151], though such intakes are unlikely to be advisable for athletes. Importantly, alcohol-free wine has also been shown to enhance endogenous antioxidant enzyme activity, including increases in glutathione reductase and superoxide dismutase, after only seven days of consumption [152]. These findings suggest that alcohol-free wine may offer a practical strategy for athletes who aim to avoid alcohol while maintaining access to polyphenolic benefits [16].

The MD may also support regulation of exercise-induced inflammation without impairing training adaptations. Greater MD adherence reduces systemic inflammatory markers, partly via epigenetic regulation and NF-κB pathway suppression [135,136]. Antioxidant-rich MD components, including fruits, contribute to post-exercise inflammation attenuation [138,139]. The diet’s anti-inflammatory properties also counteract age-related chronic inflammation (“inflammaging”) [153,154].

Immune function is an additional consideration for athletic performance, as some evidence suggests that susceptibility to infection may increase after strenuous training blocks or competitive events. Upper respiratory tract infections (URTI) are particularly common and can compromise training consistency and competitive outcomes [155,156,157,158,159,160,161]. Diet exerts a substantial impact on immune cell function [162], suggesting that a nutrient-rich dietary pattern could play a preventive role. While direct evidence linking whole-diet MD adherence to reduced infection risk in athletes remains limited, population-based findings are promising. A large-scale observational study reported a 26% reduction in sepsis risk among adults with high versus low MD adherence [163]. Polyphenols, including resveratrol, show antibacterial and antiviral effects [164,165,166], leading to the hypothesis that they could serve as supportive adjunctive therapies against highly transmissible viral infections, including COVID-19 [167,168]. Evidence of antiviral effects in vivo and ex vivo, including in athletic cohorts, has also been reported [169,170,171]. Quercetin, a polyphenol found in onions, leafy greens, and wine, has received particular attention for its potential to reduce URTI symptoms after strenuous exercise. In one study, 1 g/day of quercetin reduced URTI symptom severity in the two weeks following intensive cycling [172], while a polyphenol-rich non-alcoholic beer consumed for five weeks reduced URTI incidence by more than threefold after a marathon [173]. Intriguingly, these benefits may occur independently of measurable changes in oxidative stress or immune markers, suggesting alternative mechanisms, potentially antiviral in nature [172]. However, achieving quercetin doses comparable to those used in supplementation studies through diet alone is challenging [174]. Nonetheless, these data largely derive from controlled experimental settings or supplementation studies, and translation to whole-diet MD adherence in athletic populations remains uncertain. Therefore, while mechanistically plausible, claims regarding direct infection risk reduction should be considered preliminary.

Overall, current evidence suggests that the MD, through its natural abundance of antioxidants, anti-inflammatory lipids, and polyphenols, may offer a practical and physiologically supportive dietary model for athletes. However, further research is necessary to determine optimal dosing of specific bioactive compounds and to evaluate whether the MD confers superior effects relative to other nutrient-dense dietary patterns commonly recommended in sport. In this context, nutritional recommendations for competitive athletes must inevitably differ from those for inactive individuals due to substantially higher energy expenditure and macronutrient requirements. At present, no direct evidence confirms that the physiological benefits traditionally associated with the MD are fully preserved when total energy intake is markedly increased, as typically required by athletes. However, because the MD represents primarily a qualitative dietary model—mostly centered on food choice and meal composition rather than on fixed caloric thresholds—it is plausible to hypothesize that these benefits may remain relevant even within higher-energy dietary frameworks. Accordingly, the recommendation to adopt a Mediterranean-style approach refers to the prioritization of MD-characteristic foods as the main sources of the macronutrients required by athletes, as well as to the structuring of meals in alignment with MD principles. Nonetheless, the overall calculation of energy and macronutrient needs must be carefully individualized, as these quantitative aspects differ substantially from those of inactive individuals. A qualitative Mediterranean approach therefore aims to provide athletes with the potential benefits of this dietary pattern while allowing for the quantitative adjustments necessary to meet sport-specific energy demands.

## 6. Conclusions and Future Directions

The relationship between exercise-induced oxidative stress, cellular redox signaling, and nutritional modulation is complex and dynamic. While ROS are fundamental mediators of training adaptation, uncontrolled oxidative stress can impair muscle function, prolong recovery, and compromise performance. The MD provides a biologically coherent framework for supporting redox balance, due to its high content of polyphenols, carotenoids, unsaturated fatty acids, and other bioactive compounds that act synergistically to enhance endogenous antioxidant capacity. Importantly, the MD does not merely supply exogenous antioxidants; it shapes the biochemical environment in which redox-sensitive signaling pathways operate, enabling the preservation of beneficial oxidative cues while preventing excessive tissue damage. The combination of antioxidant-rich plant foods, EVOO as a primary lipid source, and omega-3-containing seafood contributes to a sustained, long-term modulation of inflammatory tone, mitochondrial efficiency, and cellular resilience. These characteristics suggest potential benefits for athletic training, where maintaining a balance between adaptation and recovery is essential. However, current evidence in athletes remains limited and largely based on observational or cross-sectional studies, which show considerable heterogeneity in study populations, methodologies, and dietary assessments. Controlled, athlete-centered interventions are needed to clarify the effects of MD adherence on performance metrics, mitochondrial adaptations, immune function, and recovery from overreaching or overtraining. Further research should also consider individual variability in diet–training interactions, including genetic factors, microbiome composition, habitual dietary patterns, and training load. Personalized strategies that adjust polyphenol intake, lipid composition, and nutrient timing according to metabolic and sport-specific demands represent a promising direction. Practical implementation challenges—such as accessibility, culinary literacy, and adherence during travel and competition—should also be addressed.

In summary, the MD emerges as a promising and biologically coherent dietary model, potentially supporting oxidative balance and exercise performance. Its emphasis on whole foods and synergistic nutrient interactions positions it as a valuable alternative to isolated antioxidant supplementation, which may interfere with adaptive redox signaling. Continued mechanistic studies, well-controlled athlete-focused interventions, and personalized nutrition approaches will be essential to translate these preliminary and heterogeneous findings into concrete performance benefits.

## Figures and Tables

**Table 2:** **Mediterranean Diet-Based Recommendations for Athletes**. Practical, evidence-based recommendations for applying Mediterranean Diet principles in athletic contexts with emphasis on antioxidant and recovery support. These recommendations were derived and adapted from Griffiths et al., 2022 [16].

Goal/Physiological Target	Mediterranean Diet Strategy	Rationale
Maintain glycogen availability	Periodize carbohydrates (6–12 g/kg/day) using whole grains, legumes, starchy vegetables.	Supports carbohydrate needs while preserving micronutrient and polyphenol density.
Optimize muscle repair and adaptation	1.2–2.0 g/kg/day protein distributed evenly across meals; include fish, poultry, legumes, dairy.	Promotes sustained MPS; MD proteins also provide antioxidant phytochemicals.
Ensure optimal fat intake	20–35% of total energy intake; prefer MUFA and PUFA food sources	Support hormonal and metabolic functions and contrast inflammation.
Enhance antioxidant defense around training	Use EVOO as primary fat	EVOO phenolics (hydroxytyrosol, oleuropein) modulate redox-sensitive signaling and support mitochondrial function.
Support inflammation resolution without blunting adaptation	Include 2–4 servings/week oily fish and daily nuts/seeds.	EPA/DHA modulate eicosanoid/NF-κB pathways and support inflammation resolution.
Immune support in heavy training blocks	Ensure daily mixed polyphenol intake; consider polyphenol-rich beverages when alcohol avoided.	Polyphenols may support mucosal immune defense and reduce URTI symptom incidence.
Support gut health while preventing GI distress in competition	Maintain habitual fiber, but reduce fermentable fiber 24–72 h pre-competition.	Preserves microbiota benefits while avoiding GI discomfort during performance.
Alcohol/wine consumption	Small-moderate wine intake is permitted with meals; consider alcohol-free polyphenol-rich beverages during recovery periods.	Wine polyphenols can increase antioxidant enzyme activity; avoiding alcohol protects recovery.

Abbreviations: muscle protein synthesis, MPS; Mediterranean diet, MD; extra-virgin olive oil, EVOO; eicosapentaenoic acid, EPA; docosahexaenoic acid, DHA; nuclear factor kappa-light-chain-enhancer of activated B cells, NF-κB; upper respiratory tract infections, URTI; gastrointestinal, GI.

## Data Availability

No new data were created or analyzed in this study.

## Abbreviations

The following abbreviations are used in this manuscript:

8OHdG	8-hydroxy-2′-deoxyguanosine
CK	Creatine kinase
CoQ10	Coenzyme Q10
EVOO	Extra virgin olive oil
GPx	Glutathione peroxidase
H_2_O_2_	Hydrogen peroxide
MAPK	Mitogen-activated protein kinase
MD	Mediterranean diet
MDA	Malondialdehyde
NAC	N-acetylcysteine
NEAC	Non-enzymatic antioxidant capacity
NF-κB	Nuclear factor kappa-light-chain-enhancer of activated B cells
Nrf2	Nuclear factor erythroid 2-related factor 2
OO	Olive oil
PGC-1α	Peroxisome proliferator-activated receptor gamma coactivator 1-alpha
PI3K/Akt	Phosphatidylinositide 3-kinase/protein kinase B
ROS	Reactive oxygen species
SOD	Superoxide dismutase
URTI	Upper respiratory tract infection
VOO	Virgin olive oil
XO	Xanthine oxidase
